# Supplementary material for: Machine learning algorithms’ application to predict childhood vaccination among children aged 12–23 months in Ethiopia: Evidence 2016 Ethiopian Demographic and Health Survey dataset
Source: PLoS One. 2023 Oct 18;18(10):e0288867. doi: 10.1371/journal.pone.0288867 (PMC10584162; doi:10.1371/journal.pone.0288867)
Supplement: S1 File — (PDF) [file pone.0288867.s001.pdf]

PLOS ONE Clinical Studies Checklist

***PLOS ONE* manuscript number:** ______________________

Complete the following if your study involved human participants or human subjects' data. These questions should be addressed for prospective and retrospective studies.

1. Did you obtain ethics approval for this study?

    - If yes, please upload (file type "Other") the original approval document you received from your ethics committee. If the original document is in another language, please also provide an English translation.
        - ✓ **N/A**
    - If you did not obtain ethical approval, please explain why this was not required.

**N/A. Because the study does not need ethical approval.**

2. If your study involved human participants, please report in the Methods section when participants were recruited to the study.

    - ✓ **The actual data collection period was written in the method section**

3. If you are reporting a study of medical records or archived samples, please report in the Methods section the date range in which human subjects' data/samples were collected and the date(s) when you conducted this study.

    - ✓ **N/A**

4. Please specify in the Methods section whether authors had access to information that could identify individual participants during or after data collection.

    - ✓ **Completed, and the detail for this issue have been written in the ethical statement section of the manuscript.**

5. If you are reporting an observational study – i.e. cohort, case-control, and cross-sectional studies – we recommend that the work is reported as per the requirements of the STROBE guidelines, and that you provide a completed STROBE checklist as a Supporting Information file with your submission.

    The STROBE checklist was developed to improve the reporting of observational human subjects research, and is available here: http://strobe-statement.org/fileadmin/Strobe/uploads/checklists/STROBE_checklist_v4_combined_PlosMedicine.docx.

    - ✓ **N/A**

6. Please ensure that the author list and Corresponding Author entered in Editorial Manager match the author list and Corresponding Author in your manuscript file.

    - ✓ **Completed**
