# Supplementary material for: Machine learning algorithms’ application to predict childhood vaccination among children aged 12–23 months in Ethiopia: Evidence 2016 Ethiopian Demographic and Health Survey dataset
Source: PLoS One. 2023 Oct 18;18(10):e0288867. doi: 10.1371/journal.pone.0288867 (PMC10584162; doi:10.1371/journal.pone.0288867)
Supplement: Attachment — Submitted filename: PONE-D-23-09622.pdf [file pone.0288867.s002.pdf]

PLOS ONE

Predicting childhood vaccination among children aged 12-23 months in Ethiopia: Using machine learning algorithms

--Manuscript Draft--

Manuscript Number:	PONE-D-23-09622
Article Type:	Research Article
Full Title:	Predicting childhood vaccination among children aged 12-23 months in Ethiopia: Using machine learning algorithms
Short Title:	Predicting childhood vaccination among children aged 12-23 months
Corresponding Author:	Addisalem Workie Demsash<br>Mettu University<br>Mettu, ETHIOPIA
Keywords:	
Abstract:	Introduction: Childhood vaccination is cost-effective public health interventions to reduce child mortality and morbidity. But, vaccination coverage remains low, and previous similar studies have not focused on machine learning algorithms to predict childhood vaccination. Therefore, knowledge extraction, association rule formulation, and discovering insights from hidden patterns in vaccination data are limited. Therefore, this study aimed to predict childhood vaccination among children aged 12-23 months using the best machine learning algorithm.<br>Methods: A total of 1617 samples of living children aged 12-23 months were used from the 2016 Ethiopian Demographic and Health Survey dataset. The data was pre-processed into two groups (70% for training, and 30% for testing), and eight machine learning algorithms were used for model building and comparison. All the included algorithms were evaluated using confusion matrix elements. Informational gain value was used to select important attributes to predict childhood vaccination among children aged 12-23 months. The If/ then logical association was used to generate rules based on relationships among attributes, and Weka version 3.8.6 software was used to perform all the prediction analysis.<br>Results: PART was the first best machine learning algorithm to predict childhood vaccination with 95.53% classification accuracy. J48, multilayer perceptron, and random forest models were the consecutively best machine learning algorithms to predict childhood vaccination among children aged 12-23 months with 89.24%, 87.20%, and 82.37% OF accuracy, respectively. Antenatal care visits, institutional delivery, visits to health facilities, mothers' higher education, and rich wealth status were the top five important attributes to predict childhood vaccination. A total of seven associated rules were generated that could probably determine the magnitude of childhood vaccination among children aged 12-23 months. Seven association rules were generated for childhood vaccination. If wealth status=3 (Rich), adequate ANC visits =1 (yes), and residency=2 (Urban), then the probability of childhood vaccination would be 86.73%. This was the first association rule for childhood vaccination.<br>Conclusions: The PART, J48, multilayer perceptron, and random forest algorithms are the top four algorithms to predict childhood vaccination. Machine learning algorithms are critical to predict child vaccination, extract knowledge, and generate rules. The findings provide an insight into how to implement child vaccination and serve as a framework for machine learning.
Order of Authors:	Addisalem Workie Demsash
Opposed Reviewers:	
Additional Information:	
Question	Response
**Financial Disclosure**<br><br>Enter a financial disclosure statement that describes the sources of funding for the	Financial supports were not recieved fro this study.

Powered by Editorial Manager® and ProduXion Manager® from Aries Systems Corporation

work included in this submission. Review the submission guidelines for detailed requirements. View published research articles from *PLOS ONE* for specific examples.<br><br>This statement is required for submission and **will appear in the published article** if the submission is accepted. Please make sure it is accurate.<br><br>**Unfunded studies**<br>Enter: *The author(s) received no specific funding for this work.*<br><br>**Funded studies**<br>Enter a statement with the following details:<br>• Initials of the authors who received each award<br>• Grant numbers awarded to each author<br>• The full name of each funder<br>• URL of each funder website<br>• Did the sponsors or funders play any role in the study design, data collection and analysis, decision to publish, or preparation of the manuscript?<br>• **NO** - Include this sentence at the end of your statement: *The funders had no role in study design, data collection and analysis, decision to publish, or preparation of the manuscript.*<br>• **YES** - Specify the role(s) played.<br><br>* typeset	
**Competing Interests**<br><br>Use the instructions below to enter a competing interest statement for this submission. On behalf of all authors, disclose any competing interests that could be perceived to bias this work—acknowledging all financial support and any other relevant financial or non-financial competing interests.<br><br>This statement is required for submission and **will appear in the published article** if the submission is accepted. Please make sure it is accurate and that any funding sources listed in your Funding Information	The author declared that there are no competing interests in this work.

later in the submission form are also declared in your Financial Disclosure statement.<br><br>View published research articles from *PLOS ONE* for specific examples.<br><br>**NO authors have competing interests**<br><br>Enter: *The authors have declared that no competing interests exist.*<br><br>**Authors with competing interests**<br><br>Enter competing interest details beginning with this statement:<br><br>*I have read the journal's policy and the authors of this manuscript have the following competing interests: [insert competing interests here]*<br><br>* typeset	
**Ethics Statement**<br><br>Enter an ethics statement for this submission. This statement is required if the study involved:<br><br>• Human participants<br>• Human specimens or tissue<br>• Vertebrate animals or cephalopods<br>• Vertebrate embryos or tissues<br>• Field research<br><br>Write "N/A" if the submission does not require an ethics statement.<br><br>General guidance is provided below. Consult the submission guidelines for detailed instructions. **Make sure that all information entered here is included in the Methods section of the manuscript.**	N/A

**Format for specific study types**<br><br>**Human Subject Research (involving human participants and/or tissue)**<br>• Give the name of the institutional review board or ethics committee that approved the study<br>• Include the approval number and/or a statement indicating approval of this research<br>• Indicate the form of consent obtained (written/oral) or the reason that consent was not obtained (e.g. the data were analyzed anonymously)<br><br>**Animal Research (involving vertebrate animals, embryos or tissues)**<br>• Provide the name of the Institutional Animal Care and Use Committee (IACUC) or other relevant ethics board that reviewed the study protocol, and indicate whether they approved this research or granted a formal waiver of ethical approval<br>• Include an approval number if one was obtained<br>• If the study involved *non-human primates*, add *additional details* about animal welfare and steps taken to ameliorate suffering<br>• If anesthesia, euthanasia, or any kind of animal sacrifice is part of the study, include briefly which substances and/or methods were applied<br><br>**Field Research**<br><br>Include the following details if this study involves the collection of plant, animal, or other materials from a natural setting:<br>• Field permit number<br>• Name of the institution or relevant body that granted permission	
**Data Availability**<br><br>Authors are required to make all data underlying the findings described fully available, without restriction, and from the time of publication. PLOS allows rare exceptions to address legal and ethical concerns. See the PLOS Data Policy and FAQ for detailed information.	Yes - all data are fully available without restriction

A Data Availability Statement describing where the data can be found is required at submission. Your answers to this question constitute the Data Availability Statement and **will be published in the article**, if accepted.<br><br>**Important:** Stating 'data available on request from the author' is not sufficient. If your data are only available upon request, select 'No' for the first question and explain your exceptional situation in the text box.<br><br>Do the authors confirm that all data underlying the findings described in their manuscript are fully available without restriction?	
**Describe where the data may be found in full sentences. If you are copying our sample text, replace any instances of XXX with the appropriate details.**<br><br>• If the data are **held or will be held in a public repository**, include URLs, accession numbers or DOIs. If this information will only be available after acceptance, indicate this by ticking the box below. For example: *All XXX files are available from the XXX database (accession number(s) XXX, XXX.*).<br>• If the data are all contained **within the manuscript and/or Supporting Information files**, enter the following: *All relevant data are within the manuscript and its Supporting Information files.*<br>• If neither of these applies but you are able to provide **details of access elsewhere**, with or without limitations, please do so. For example:<br><br>*Data cannot be shared publicly because of [XXX]. Data are available from the XXX Institutional Data Access / Ethics Committee (contact via XXX) for researchers who meet the criteria for access to confidential data.*<br><br>*The data underlying the results presented in the study are available from (include the name of the third party*	All relevant data are within the manuscript and its Supporting Information files.

and contact information or URL).<br>• This text is appropriate if the data are owned by a third party and authors do not have permission to share the data.<br><br>* typeset	
Additional data availability information:	

Powered by Editorial Manager® and ProduXion Manager® from Aries Systems Corporation

Dear Plos one journal

This is to submit a manuscript entitled ***Predicting childhood vaccination among children aged 12-23 months in Ethiopia: Using machine learning algorithms*** for consideration of publication in the journal.

This manuscript might be important for addressing information or research gap about predicting childhood vaccination. The findings might be critical for health policymakers for immunization coverage and reduction of vaccine dropout. This manuscript is in line with the journal's scope and objective.

The manuscript was not submitted and is not under consideration in another journal.

If there is anything not well addressed, please let us know and learn from the journal editor and reviewers. For further communication please contact the corresponding authors via addisalemworkie599@gmail.com.

With kind regards!!

Thank you!!

Manuscript
Click here to access/download;Manuscript;Prediction child vaccination.docx

Predicting childhood vaccination among children aged 12-23 months in Ethiopia: Using machine learning algorithms

Addisalem Workie Demsash[a*]

[a]Mettu University, College of health science, department of health informatics, Mettu, Ethiopia

***Correspondence author:** addisalemworkie599@gmil.com

Abstract

Introduction: Childhood vaccination is cost-effective public health interventions to reduce child mortality and morbidity. But, vaccination coverage remains low, and previous similar studies have not focused on machine learning algorithms to predict childhood vaccination. Therefore, knowledge extraction, association rule formulation, and discovering insights from hidden patterns in vaccination data are limited. Therefore, this study aimed to predict childhood vaccination among children aged 12-23 months using the best machine learning algorithm.

Methods: A total of 1617 samples of living children aged 12-23 months were used from the 2016 Ethiopian Demographic and Health Survey dataset. The data was pre-processed into two groups (70% for training, and 30% for testing), and eight machine learning algorithms were used for model building and comparison. All the included algorithms were evaluated using confusion matrix elements. Informational gain value was used to select important attributes to predict childhood vaccination among children aged 12-23 months. The If/ then logical association was used to generate rules based on relationships among attributes, and Weka version 3.8.6 software was used to perform all the prediction analysis.

Results: PART was the first best machine learning algorithm to predict childhood vaccination with 95.53% classification accuracy. J48, multilayer perceptron, and random forest models were the consecutively best machine learning algorithms to predict childhood vaccination among children aged 12-23 months with 89.24%, 87.20%, and 82.37% OF accuracy, respectively. Antenatal care visits, institutional delivery, visits to health facilities, mothers' higher education, and rich wealth status were the top five important attributes to predict childhood vaccination. A total of seven associated rules were generated that could probably determine the magnitude of childhood vaccination among children aged 12-23 months. Seven association rules were generated for childhood vaccination. If wealth status=3 (Rich), adequate ANC visits =1 (yes), and residency=2 (Urban), then the probability of childhood vaccination would be 86.73%. This was the first association rule for childhood vaccination.

Conclusions: The PART, J48, multilayer perceptron, and random forest algorithms are the top four algorithms to predict childhood vaccination. Machine learning algorithms are critical to predict child vaccination, extract knowledge, and generate rules. The findings provide an insight into how to implement child vaccination and serve as a framework for machine learning.

Keywords: Child, Vaccination, Prediction, Machine learning

Introduction

Globally, nearly 44% of child deaths occurred under 28 days of birth [1]. Around 75% of child deaths occur within 12 months of birth, and an estimated 4.1 million infants are projected to die in 2017 [2]. The rate of child deaths in developing countries is the highest in the world [2, 3]. Around 1.2 million children are predicted to have died in Africa in the first 28 days of birth [4], and nearly 49% of child deaths are predicted to have occurred in Sub-Saharan countries [3]. According to the World Health Organization (WHO), more than half of child deaths are caused by infectious diseases that are easily preventable and treatable through simple and affordable interventions [5]. Worldwide, childhood mortality and morbidity are caused by tuberculosis, diphtheria, pertussis, tetanus, polio, and measles [6].

Child deaths due to diphtheria, pertussis, tetanus, polio, and measles are easily preventable through vaccines. Childhood vaccination is one of the most successful and cost-effective public health interventions for common childhood illnesses like pneumonia, diphtheria, tetanus, whooping cough, and measles [7]. Nowadays, nearly 3 million child deaths due to diphtheria, tetanus, whooping cough, and measles are prevented through child vaccination [8]. Over the past decade, more than 1,000,000 children's lives have been saved by immunization programs, and infectious and communicable diseases have been controlled through child vaccination [9].

Nonetheless, 12.9 million children did not receive recommended vaccines across the world [8]. Sufficient numbers of children did not complete their immunization schedule due to various challenges and barriers [10]. Nearly 21 million of children have been projected to miss out on vaccines, and two third of vaccination missing were occurred in developing regions due to outbreak of new case [11]. Nearly 21 million children have been projected to miss out on vaccines, and two-thirds of the missing vaccinations occurred in developing regions due to an outbreak of new cases [12]. In Ethiopia, infant vaccination doses were usually delayed, with 63.8 percent of Diphtheria Pertussis Tetanus (DTP) dose 1, 63.1 percent of Polio dose 1, and 68.5 percent of measles delivered after the recommended date [13]. According to the Ethiopia Demographic and Health Survey (EDHS), data on vaccination coverage among children aged 12–23 months who received specific vaccines at any time prior to the survey revealed that only four out of ten children (43%) had received all basic vaccinations [14]. According to the WHO, the mean dropout rates of Bacillus Calmette–Guérin (BCG) and measles are 34.6% and 28.6%, respectively [15].

According to a traditional and multilevel logistic regression analysis report, different factors are reported that could affect child vaccination and immunization coverage. Maternal education, knowledge of mothers about the vaccines and their schedule, maternal age, fear of side effects, having antenatal care (ANC) visits, and giving birth at a health institution are some of the maternally related factors that affect childhood vaccinations [16-19]. Additionally, availability of vaccines, migration of caregivers, household income level, and sex of household heads are factors that affect childhood vaccination status [20, 21]. Moreover, the sex and age of children, their birth interval and order, multiple children born at a time, a mother's media exposure, being a rural resident, and having distant health facilities are also factors associated with childhood vaccination [22, 23]. However, the odds ratio and relative risk of traditional and multilevel logistic regression do not meaningfully classify attributes and do not discover new insight [24].

Despite the efforts of the government to improve child vaccination, increase vaccination coverage, and reduce vaccine dropout rates, vaccine providers and health programmers lack available on-site information handling tools to target high-risk children for vaccine dropout, late and incomplete vaccination [15]. Therefore, low-income countries would model and visualize the childhood vaccination risks on large datasets to identify attributes for childhood vaccination and target children who are at a high risk of dropping out or delaying the next vaccine dose.

Massive amounts of biomedical and public health data are categorized, and predicted using a variety of predictive algorithms to gain new knowledge and reveal hidden relationships and trends [25]. Multidimensional data mining techniques were used to correctly forecast future immunization outcomes based on existing data, and to predict features of typical childhood immunization schedules [26]. Predictive analytics tools are potent and widely applicable for learning. Numerous machine learning algorithms have reportedly been used in earlier studies to predict disease prevalence, the use of healthcare services, vaccination uptake [27], routine immunization [15], childhood vaccination and mortality [28, 29]. For automated detection, identifying connections that aren't leaner, and identifying significant patterns in data, machine learning algorithms are essential [30].

Specifically, random forests, logistic regression, J48, logit boost, and Addaboost algorithms were used to predict under five and neonatal mortality [29, 31], undernutrition status of children [32], malnutrition among children [33, 34]. Additionally, Naïve Bayes and PART algorithms

are also used to forecast and classify text documents [35]. Prediction of childhood vaccination based on machine learning techniques is insufficient. Currently, massive amounts of data are being generated. So, these must be presented with the best data analysis tools. Policy makers and stakeholders need accurate predictions on various aspects of immunization and other health parameters for effective actions. Researchers are needed to test and compare various prediction and classification algorithms that are needed to classify and predict childhood vaccination. Therefore, this study aimed to: **(1)** evaluate different machine learning algorithms using model evaluation matrix parameters; **(2)** identify important attributes for childhood vaccination based on the best performance algorithm; and **(3)** generate an association rule that together determines the vaccination of children aged 12-23 months in Ethiopia.

Methods and materials

Study design and setting

A cross-sectional study design was conducted across the nine regions of Ethiopia. Ethiopia is located in the Horn of Africa and is bordered by Eritrea to the North, Djibouti and Somalia to the East, Sudan and South Sudan to the West, and Kenya to the South. Ethiopia has nine regional states with two administrative cities. These are subdivided into different administrative zones (817 Woredas and 16253 Kebeles).

Data source

The 2016 Ethiopian Demographic and Health Survey (EDHS) dataset was used from the DHS program website (https://dhsprogram.com). The survey was conducted by the Ethiopian Public Health Institute (EPHI) in collaboration with the Central Statistical Agency (CSA). The actual data collection period was conducted from January 18, 2016, to June 27, 2016.

Sampling techniques and procedures

The sampling frame used for the 2016 EDHS is a frame of all Census Enumeration Areas (EAs) created for the 2016 Ethiopia Population and Housing Census (EPHC) and conducted by the Central Statistical Agency (CSA). The census frame is a complete list of the 84, 915 EAs, covering an average of 181 households, created for the 2016 EPHC. The sample for the 2016 EDHS was designed to provide estimates of key indicators for the country as a whole, for urban and rural areas separately, and for each of the nine regions and the two administrative cities. Two-stage stratified cluster sampling was used. Each region was stratified into urban and rural

areas. In the selected EAs, a household listing operation was done, and the results were used as a sampling frame for household selection in the second stage. Finally, a fixed number of households per cluster was selected. Samples of EAs were selected independently in each stratum through implicit stratification and equal proportional allocation.

Study populations

In this study, all living children aged 12-23 months were the source population, and all sampled living children aged 12-23 months living with their mother were the study population. Details about the methodology of the data source, sampling procedure and source population were presented in 2016 EDHS report [36].

Study variables

Dependent variable

Childhood vaccination among children aged 12-23 months.

Independent variables

Socio-demographic characteristics of households, such as wealth status, educational status of mothers, age of mother, region, residency, sex and age of children, birth interval and birth order, sex of households' heads, ANC visit, place of delivery, working status, visiting health facility, and media exposure were used as independent attributes to predict childhood vaccination mong children aged 12-23 months in Ethiopia.

Operationalizations

Childhood vaccination

Childhood vaccination among children aged 12–23 months was assessed using one dose of BCG, three dose of polio vaccine, three doses of DPT vaccine, and one dose of measles vaccine. Accordingly, the children had basic childhood vaccination if the children received at least one dose BCG vaccine, three doses of the polio vaccine, three doses of DPT vaccine, and one dose of measles vaccine, else children had not received basic childhood vaccination. Information on basic childhood vaccination status was obtained (1) written vaccination cards, (2) the mothers' verbal reports, and (3) health facility records [36].

Birth interval

The period between two successive live birth is birth interval. For this study, a birth interval of less than <33 months between two consecutive live births is **short birth interval**, whereas birth interval of 33 and above is an **optimum birth interval [37]**.

ANC visit

The pregnant women had visited a health facility during their pregnancy for ANC services. Accordingly, the women had adequate ANC visits when the women visited the health facility at least four times for ANC services, otherwise inadequate ANC visit [38, 39].

Media exposure

If the mothers had access of either radio or television or both, then the mothers were had media exposure; otherwise unexposed to media [40].

Data management and statically analysis

Data cleaning and labelling were performed using STATA version 15 software to prepare the data for analysis. Variables were recoded to meet the desired classification. To ensure the representativeness of survey results at the national level [41], sampling weights were applied during the analysis. The STATA version 15 software was used for data management and logistic regression analysis. Weka version 3.8.6 software was used for data pre-processing, important attribute selection that could predict childhood vaccination and generating rules associated with the childhood vaccination.

Ethical approval and consent to participate

Ethical clearance was not necessary for this study since it was based on publicly available data sources. Informed consent from the study participants was also not applicable to this study. There are no attributes that uniquely identify individuals or households in this study. As a result, specific ERs, individuals, and households cannot be identified uniquely (**S1File)**.

Data pre-processing

Data pre-processing was used to remove missing and incomplete records. In the dataset, noise, outliers, and inconsistency are common. Therefore, all these unnecessary data values, including duplicate variables, were removed from the data set. At this stage, all strings and categorical variables were transformed into nominal data types.

Feature selection

In this study, there were two stages of variable selection in machine learning algorithm. At the first stage, a traditional logistic regression analysis was employed for feature or independent variable selection. A variable with a p-value of less than 0.05 with backward stepwise logistic regression analysis was selected as a candidate for further important attribute selection. In the second stage, a best-performance machine learning algorithm with information gain values was used to find important features or attributes that have a major contribution to predict childhood vaccination among children aged 12-23 months in Ethiopia. The highest information gain value of an independent attribute is the most important attribute to predict the childhood vaccination. Then the next important attributes were selected based on their order of highest information gain value.

Model building

Data split and model selection

In this step, the datasets were splitted as training and testing data sets. A 7:3 classification rules were considered for training and testing dataset classification and model building. A total of 1617 instances/ observations were included to predict childhood vaccination. From a total of 1617 observations, 1132 observations (70% of total observations) were used as training dataset, and the remaining 485 observations (30% of total observations) were used as testing dataset. Training and testing data sets were used for model classification and evaluation, respectively. Various machine learning algorithms were used to predict child mortality and health service utilization [25, 33, 34]. The various appropriate machine learning algorithms such as Naïve Bayes (NB), PART, logistic regression (LR), multilayer perceptron, J48, logit boost, random forest (RF), and addaboost were used to predict childhood vaccination among children aged 12-23 months in Ethiopia.

Naïve Bayes (NB)

Naïve Bayes algorithm is a supervised machine learning algorithm, which is based on Bayes theorem and used for classification and prediction of problems. In Naïve Bayes algorithm, attributes are conditionally independent for the target class [25]. Naïve Bayes has a computational efficiency that number of attributes and classification time is linear with number of attributes, and not affected by training time. Naive Bayes algorithms had an incremental learning behaviour, could directly predict patterns with low variance, and its performance is measured by confusion matrix elements [42].

PART

PART is a hybrid approach of rule-based classification algorithm, and it uses separate and conquer classification process [35]. It creates a partial decision tree from all the iterations and considers the suitable leaf into a rule. So, it is best for performing if/ then rules to extract and build knowledge for childhood vaccination [43].

Logistic regression (LR)

Logistic regression is type of regression model which is important to model the categorical dichotomous outcome variable or feature. LR is a statistical model used to classify and predict different parameters in health [44]. It might be a binary (Binary logistic) and (multiple) model used to predict binary (multiple) outcome variable. LR has different assumptions, of which the target variable is dichotomous, and independent variables that affect the target variable are independent with each other [45].

J48 classifier algorithm

A J48 classifier algorithm is one of the best machine learning algorithms that examine categorical data based on top down recursive divide and conquer strategy [46]. J48 classifier is a simple C4.5 decision tree for classification to creates a binary tree. The algorithm is crucial for classifying the problems, and the J48 algorithm is important to ignore the missing values and able to predict for the item of missing value based on what is known about the records of another attribute. The process is to divide the available data into range based on the attribute values for that item that are found in the training data, and then classification done and rules are generated from the attributes [47].

Random forest (RF)

A random forest is a supervised machine learning algorithm used to classify and predict health problem and health service utilization [48]. RF is fastest to train and work with subsets of features. RF is important to detect complex relationships, including nonlinear and high-order interactions, and yield smallest prediction errors [49].

Addaboost and logit boost

Addaboost is an ensemble meta-learning method that enhance efficiency of binary classification tree. Addaboost uses an iterative approach to learn from the mistakes of weak classifiers, and turn them into strong ones [50, 51]. Ada Boosting is curtail to boost the

performance of decision trees based on binary classification problems [52]. Another very powerful boosting classifier algorithm (**logit boost**) was used to predict childhood vaccination in this study. **Logit boost** algorithm is designed as an alternative solution is to address the limitations of Addaboost in handling noise and outliers [53]. The overall knowledge flow of model building for data processing, analyzing and visualizing are presented in **figure 1**.

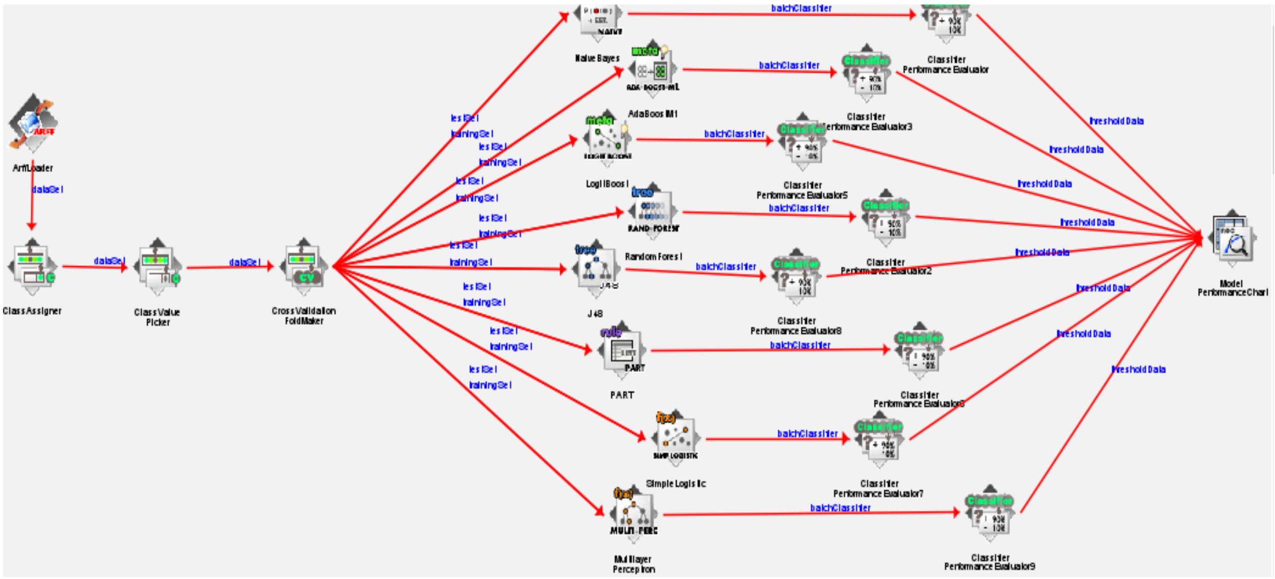

Figure 1: Knowledge flow of the models

Model evaluation

The performance of all the included algorithms has been evaluated using the confusion matrix. The accuracy of actual and predicted classes has been visualized by the confusion matrix model [54]. The predicted and actual classifications of under-five child mortality were compared using confusion matrix elements, such as true positive (TP), false positive (FP), true negative (TN), and false-negative (FN). The receiver operators' curve (ROC) was also used for model evaluation based on sensitivity, and specificity relationships. Since ROC is based on probability, the area under the ROC curve (AUC) is crucial to representing the degree or measure of separability. It tells how much the model is capable of distinguishing between classes. Hence, the higher the AUC, the better the model is at predicting true classes as true and false classes as false. Usually, the AUC value is good if it is greater than 80%, fair if it is between 70% and 80%, poor if it is between 60% and 70%, and failed if it is less than 60% [55]. A metric of interrater agreement i.e. kappa statistics was used to measure the degree of agreement/ inter rater reliability, and used to evaluate the accuracy of a classification. If Kappa statistics value is ≤ 0 indicating the agreement is worse than random agreement, 0.01-0.20

slight agreement, 0.21-0.40 fair agreement, 0.41-0.60 moderate agreement, 0.61-0.80 substantial agreement, and 0.81-1.00 almost perfect agreement [56].

The formula for the confusion matrix's element is presented in **box 1**.

Box 1: Formula for the element of confusion matrix

$$\textbf{Accuracy} = (\mathrm{TP} + \mathrm{TN})/(\mathrm{TP} + \mathrm{TN} + \mathrm{FP} + \mathrm{FN})$$

$$\textbf{Sensitivity} = \mathrm{TP}/(\mathrm{TP} + \mathrm{FN})$$

Not that Sensitivity=Recall=True Positive Rate (**TPR**)

$$\textbf{Specificity} = \mathrm{TN}/(\mathrm{TN} + \mathrm{FP})$$

$$\textbf{False positive rate} = \mathrm{FP}/(\mathrm{FP} + \mathrm{TN})$$

$$\textbf{F\_measure} = 2\mathrm{TP}/(2\mathrm{TP} + \mathrm{FP} + \mathrm{FN})$$

$$\textbf{Precision} = \textbf{Postive predictive value} = \mathrm{TP}/2\mathrm{TP} + \mathrm{FP})$$

True positive (TP): The model correctly predicts positive class of response outcome.

False positive (FP): The model incorrectly predicts positive class in the response outcome.

True negative (TN): The model correctly predicts negative class in the response outcome.

False-negative (FN): The model incorrectly predicts negative class in the response outcome.

Sensitivity: Sensitivity is the test to measure correctly positive predicted events out of a total number of positive events, and it show that the value of how many positives are predicted out of total positive classes.

Specificity: Specificity is the proportion of real negative cases that got predicted as the negative. This indicate that there will be another proportion of real negative cases, which would get predicted as positive and could be termed as false positives.

Precision: Precision is a positive predictive value, and it is the correct events divided by the total number of positive events that the classifier predicts.

F_measure: F measure is the inverse relationship between accuracy and recall. The higher value of the F-measure score predicts a better model.

Prediction and association rule mining

Once the model is built and its performance assessed, the death of children before their fifth birthday is predicted based on the independent predictors. Important variables selected based on a best-performance model were used to predict child mortality before their fifth birthday.

Although important variables are used to predict child deaths before their fifth birthday, the predictive model does not show which nominal variables are jointly associated with child deaths before their fifth birthday.

Therefore, association rule mining analysis (the **If** (antecedent)/ **then** (consequent) statements) is used to discover relationships between seemingly independent relational attributes. Association rule mining analysis is important for non-numerical and categorical types of data attributes. It is important to observe frequently occurring patterns, and identify the dependencies between attributes by supporting how frequently the if/then relationship appears in the observations, and confidence in the number of times the relationships have been found to be true. The if/ then association rule mining analysis is critically important to select important features that jointly determine under five child mortality and is the easiest way to interpret [57].

For the association rule mining analysis, apriori algorithm method was used to identify strong and frequently related attributes. The **If then** association rule is the pair of X and Y (X, Y) attributes expressed as X->Y, where X is an antecedent and Y a consequent that is as X happen Y is would also happen [58]. These rules are critically important for the prevention and control of health problems and crucial for health policymakers' proactive decision-making purposes. Various studies had widely used if/then rules in healthcare research, such as predicting childhood care and child mortality [59], predicting parasite infection [60], the pattern of new cases and stroke [61, 62], and maternal healthcare service utilization and service discontinuation [63] to identify important features. The relationship between X and Y attributes is expressed in the following way [62].

If the left attribute >**1** | X and Y positively associated to determine under five child mortality.
if the left attribute <**1** | X and Y negatively associated to determine under five child mortality.

If the left attribute=**1** | No relation between X and Y to determine under five child mortality.

The detail of data preparation, model building, important variable selection, and analysis work flow is presented in **figure 2**.

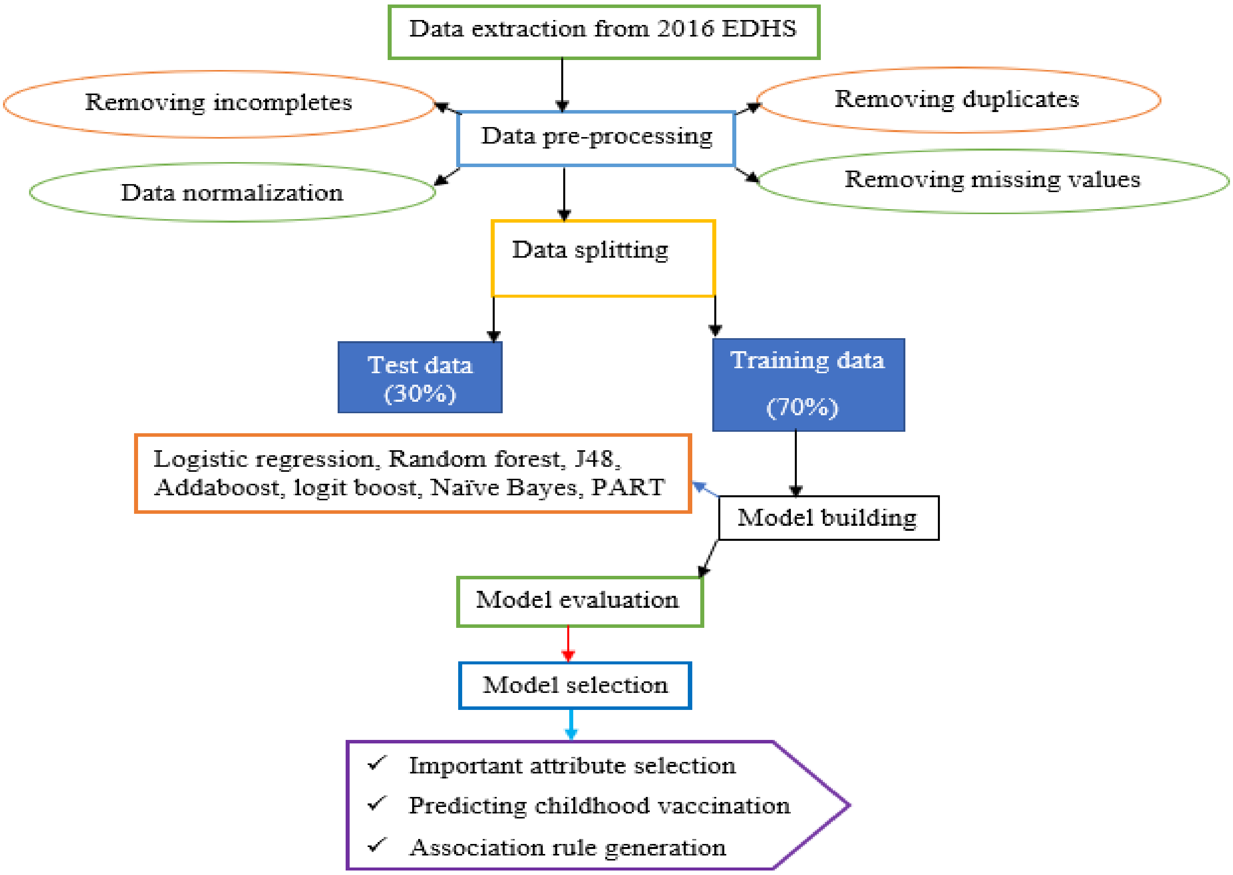

Figure 2: Work flow for data pre-processing, and child death prediction processing.

Results

Sociodemographic characteristics of the study participants

A total of 1617 weighted sample of children aged from 12-23 months were included for analysis. Three majority (62.52%) of children were born form mothers aged above 35 years. The mean age of mothers was 3.53. The majority (72.5%) of children were born from mothers who had not had formal education. Seven hundred thirty (45.1%) and two hundred eighty-eight (17.8%) of children were from Oromia and Amhara regions, respectively. The majority (91.2%) of the children were born from rural residents' mothers. Nearest to five hundred fifty-three (34.2%) and four out of ten (40.3%) of children were born from mothers whose religious were Orthodox and Muslim, respectively. Seven hundred sixty (47%) of children' mothers were poor. Nearest to half (52.9%) and majority (86.2%) of children and households heads were female and male, respectively. Six hundred seventy-five (41.7%) of children were under the age of 12-15 months, and their mean age was 1.844 (**Table 1**).

Table 1: Socio-demographic characteristics of study participant using 2016 EDHS data (n=1617).

Variable	Category	Frequency (n)	Percent (%)
Mothers' educational status	No formal education	1172	72.5
	Primary	377	23.3
	Secondary	39	2.4
	Higher	29	1.8
Region	Tigray	116	7.2
	Afar	16	1.0
	Amhara	288	17.8
	Oromia	729	45.1
	Somali	60	3.7
	Benishangul	18	1.1
	SNNPR	347	21.4
	Gambela	4	.2
	Harari	3	.2
	Addis Ababa	29	1.8
	Dire Dawa	7	.4
Mothers' age (year)	15-34	1011	62.52
	>=35	606	37.48
Family's wealth index	Poor	760	47.0
	Middle	339	20.9
	Rich	518	32.1
Mother/caregiver religion	Orthodox	553	34.2
	Catholic	26	1.6
	Protestant	346	21.4
	Muslim	652	40.3
	Traditional, and other	40	2.5
Place of residency	Urban	142	8.8
	Rural	1475	91.2
Sex of children	Male	761	47.1
	Female	856	52.9

Sex of household head	Male	1394	86.2
	Female	223	13.8
Current age of children	12-15 months	675	41.7
	16-19 months	519	32.1
	20-23 months	423	26.2

Children's and mothers' characteristics

Less than half (47.2%) of children were visited health facility in the last 12 months after birth, and the majority (70.6%) of children's mother had not work during the time of interview. Only 29.8% of children's mother had media exposure, and 29% of mother had gave birth health institutions. The majority (70.6%) of the mothers had not adequate ANC visits during their pregnancy period. The majority (64.5%) of children had birth order less than five, and 65.1% of children had an optimal birth interval (**Figure 1**).

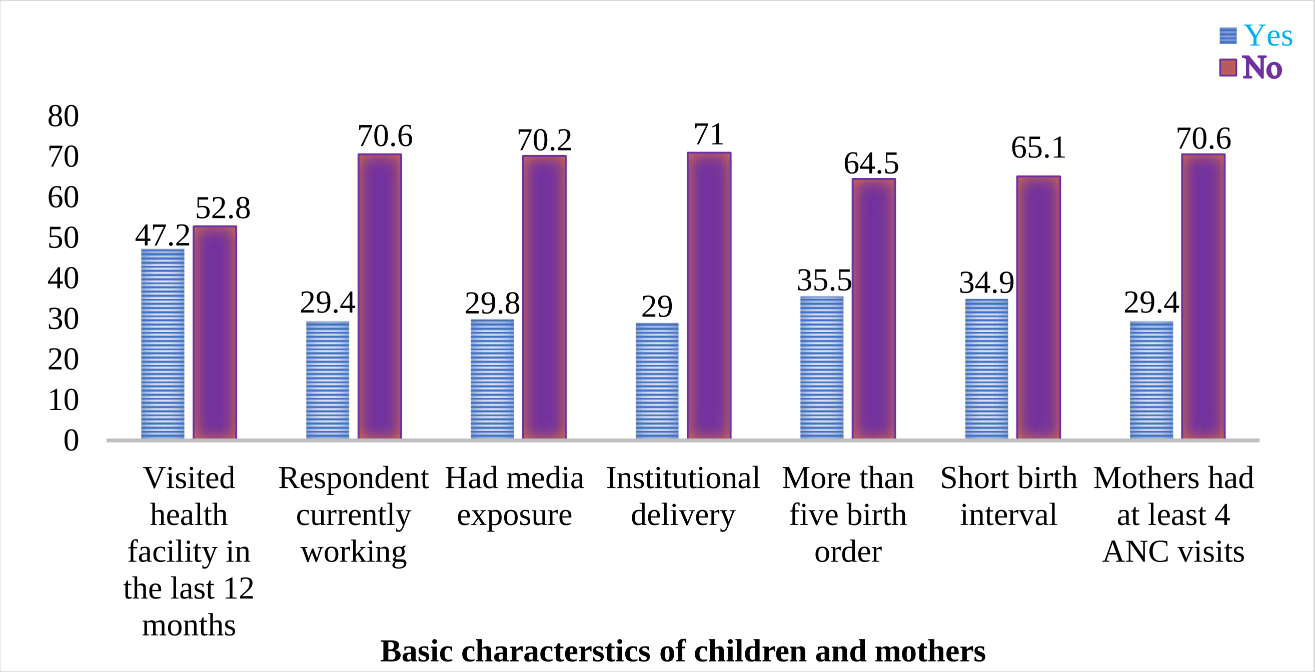

Figure 1: Children's and mothers' characteristics

Vaccination coverage among children aged 12-23 months in Ethiopia

The overall vaccination coverage of children aged from 12-23 months was 38.7%. Specifically, more than half (54.1%) of children received measles vaccination, and seven out of ten (68.6%) of children received BCG vaccine. The majority (72.9%), nearly two-third (65.2%), and more than half (53%) of children received DPT1, DPT2, and DPT3 vaccines respectively. The

majority (80.8%), nearly seven out of ten (72%), and more than half (55.9%) of children aged 12-23 months received POLIO 1, POLIO 2, and POLIO 3 respectively (**Figure 2**).

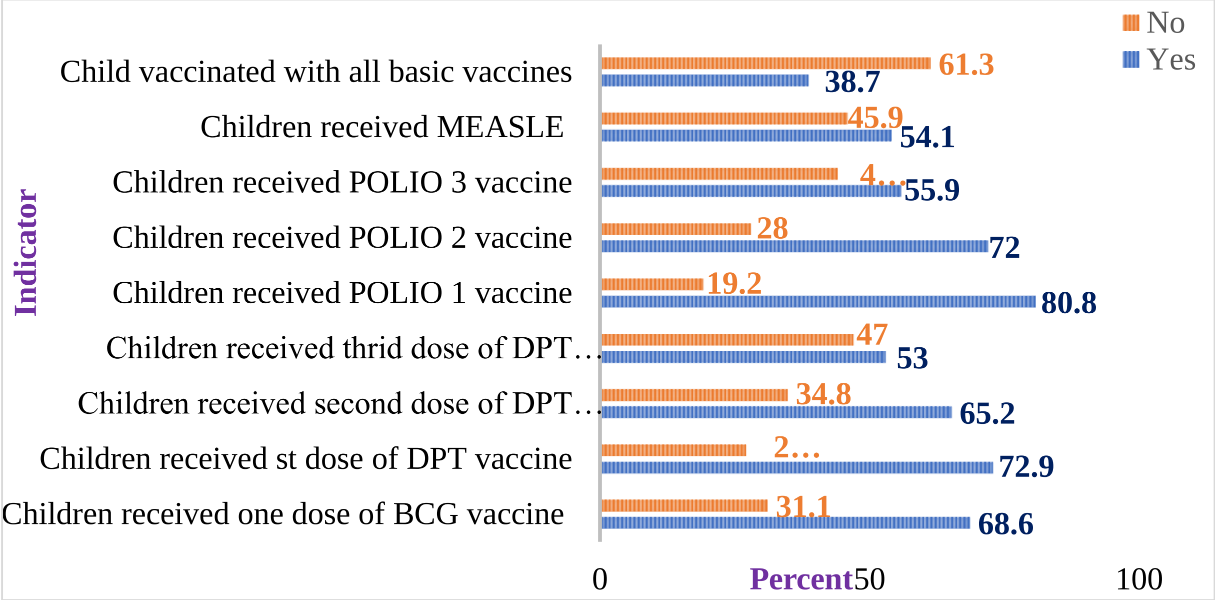

Figure 2: Children aged 12-23 months vaccinated with recommended vaccination types

Models performance to predict childhood vaccination in Ethiopia using 2016 EDHS data

Eight machine learning algorithms were used to predict childhood vaccination in Ethiopia. The PART, Naïve Bayes, logit boost, J48, random forest, addaboost, logistic regression, and multilayer perceptron algorithms were included to predict childhood vaccination. The confusion matrix parameter elements (TPR, FNR, precision, F-measure, AUR, accuracy) were used to evaluate the performance of the included algorithms. Accordingly, the PART algorithm was the first best performance algorithm to predict childhood vaccination with 95.53& of accuracy, and 91.89% of AUC. The Kappa statistics value also confirmed that the classification accuracy of the PART algorithm was almost perfect with 86.57%. The j48 algorithm was the second-best machine learning algorithm to predict childhood vaccination with 89.24% of accuracy. The 86.01% of AUR value also confirmed that j48 algorithm was best model next to the PART algorithm, and the classification accuracy of j48 algorithm had substantial agreement with 79.27% of kappa statistics. The overall machine learning algorithms comparison for childhood vaccination were presented in **table 2** and **Figure 3**.

Table 2: Model accuracy of the include machine leaning algorithms.

	**The included machine learning algorithms**

**Confusion matrix Parameters (%)**	PART	Naïve Bayes	Random forest	Logit boost	J48	Addaboost	Multilayer perceptron	LR
True positive rate (%)	89.90	64.00	88.50	72.10	88.40	69.80	83.60	70.90
False positive rate (%)	18.20	30.80	1.50	38.90	32.40	35.21	12.10	36.30
Specificity								
Precision (%)	93.80	73.90	87.80	70.60	76.00	72.00	81.00	71.70
F-measure (%)	94.30	68.20	88.60	71.30	77.71	70.90	82.30	71.30
Relative absolute error (%)	51.78	75.33	34.05	83.68	75.05	85.10	23.19	84.21
AUC (%)	91.89	72.30	82.70	73.20	86.01	72.90	83.29	65.60
Kappa statistics (%)	86.57	32.66	78.68	33.26	79.27	36.50	72.02	35.00
Accuracy (%)	95.53	66.29	82.37	77.28	89.24	68.60	87.20	65.80

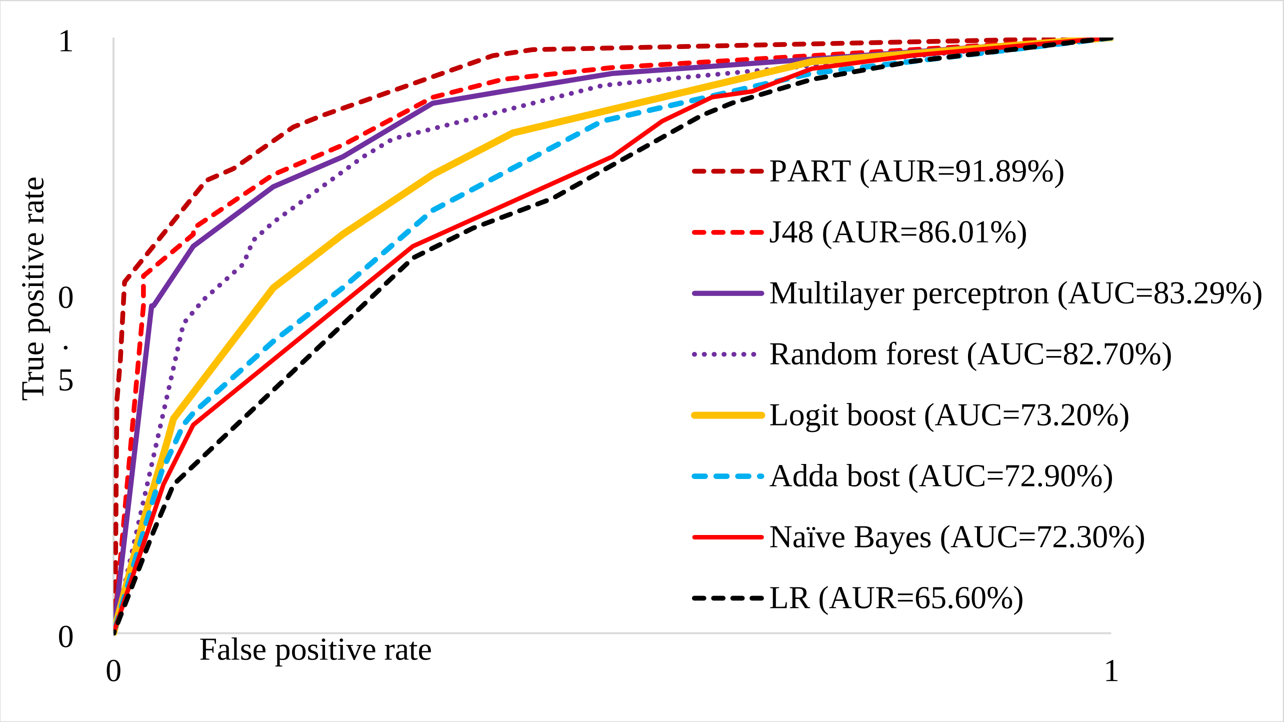

Figure 3: Comparison the eight machine learning algorithms using AUR value.

Importance attributes of childhood vaccination in Ethiopia

The information gain coefficients with 10-cross fold validation process were used to select important attributes of childhood vaccination in Ethiopia. The best performance model (PART

algorithm) was considered to select important attributes. According to the PART algorithm report, having adequate ANC visit, institutional delivery, visiting health facilities in the last 12 months, higher educational status of mothers, children whose mother were rich, being of urban resident, female household heads, mothers' age greater than 35 years, having birth order less than five, and mothers currently working were important attributes for childhood vaccination among children aged 12-23 months. The important attributes and their information gain values were presented in **table 3 and figure 3**.

Table 3: Information gain value for each predictor variables

Predictor variables	Type	Measurement	Information gain value
Adequate ANC visit, (Yes)	Nominal	Scale	0.087
Institutional delivery, (Yes)	Nominal	Scale	0.084
Visited HF in the last 12 month, (Yes)	Nominal	Scale	0.076
Educational status, (Higher)	Nominal	Scale	0071
Wealth status, (Rich)	Nominal	Scale	0.071
Residency, (Urban)	Nominal	Scale	0.062
Household sex, (Female)	Nominal	Scale	0.059
Mothers' age, (>35 years)	Nominal	Scale	0.037
Birth order >5, (No)	Nominal	Scale	0.023
Mothers' currently working, (Yes)	Nominal	Scale	0.023

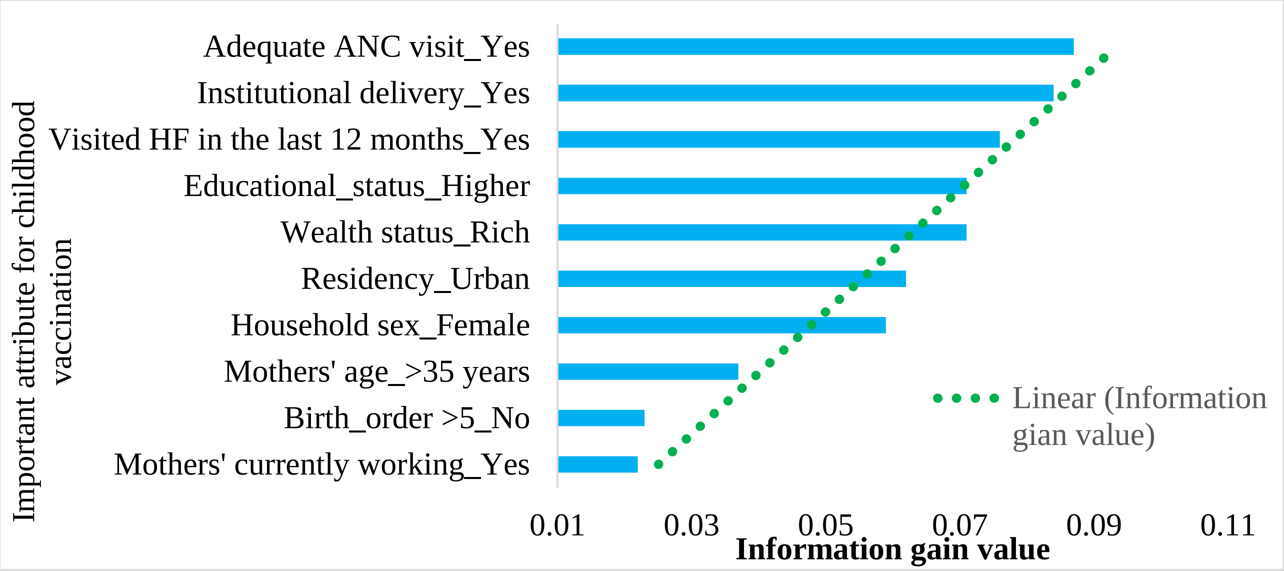

Figure 3: Important attributes to predict childhood vaccination

Association rule building

The association rule generation process was done based on important attributes selected by bet performing machine learning model. A total of seven association rules were generated, and the details of the rule were presented in box 1.

Box 1: Association rule generation and knowledge extraction

Rule 1: If wealth status=3 (Rich), adequate ANC visits =1 (Yes), and residency=2 (Urban), **then** the probability of childhood vaccination would be 86.73% (left=1.87).

Rule 2: If institutional delivery=1 (Yes), mothers' educational status=4 (Higher), and household head sex=0 (Female), **then** the probability of childhood vaccination would be 82.14% (left=1.67).

Rule 3: If adequate ANC visit=1 (Yes), mothers' age =1 (>35 years), and institutional delivery=1 (Yes), **then** the probability of childhood vaccination would be 79.21% (left=1.47).

Rule 4: If birth order >5=0 (No), visited HF in the last 12 months=1 (Yes), residency= 2 (Urban) and mothers' current working=1 (Yes), **then** the probability of childhood vaccination would be 66.81% (left=1.32).

Rule 5: If institutional delivery=1 (Yes), mothers' wealth status=2(Middle), and visited HF in the last 12 months=1 (Yes), **then** the probability of childhood vaccination would be 62.45% (left=1.25).

Rule 6: If residency=2 (Urban), birth order >5=1 (Yes), wealth status=2 (Middle), and adequate ANC visits=1 (Yes), **then** the probability of childhood vaccination would be 57.16% (left=1.17).

Rule 7: If mothers' educational status=3 (Secondary), institutional delivery=1 (Yes), and mothers currently working=1 (Yes), **then** the probability of childhood vaccination would be 51.92% (left=1.12).

Discussion

For this study, the 2016 EDHS dataset was used, with a total of 1617 observations. A 70% and 30% of total observations were used as training, and testing data sets, respectively. The aim was to evaluate machine learning algorithms using the confusion matrix element, and select important attributes that could accurately predict childhood vaccination among children aged

12-23 months in Ethiopia. Accordingly, eight machine learning algorithms were included to achieve the study objectives.

The included eight machine learning algorithms were evaluated and compared by their classification accuracy and AUR score values. Hence, the accuracy and AUR value of the PART algorithms were 95.53% and 91.89% with 10-fold cross validations, respectively. Hence, the PAR algorithm was the first accurate model to predict childhood vaccination among children aged 12-23 months. This finding was agreed with study done about data classification and terms of association [35]. The j48, multilayer perceptron, and random forest algorithms were the second, third and fourth best machine learning algorithm to predict childhood vaccination with 89.24%, 87.20%, and 82.37% of accuracy, respectively. This finding was supported by various studies conducted to predict under-five child mortality [29, 32, 64]., contraceptive discontinuation [63], stunting and malnutrition among children [65-67].

The second objective of the study was to select important attributes that could predict childhood vaccination among children aged 12-23 months in Ethiopia. From the attributes selected to predict childhood vaccination, adequate ANC visits, deliveries at health facilities, visits to HF, higher education of mothers, rich wealth status, children from urban areas, female household heads, a mother's age greater than 35 years, a child's birth order less than five, and mothers currently working were important attributes to predict vaccination of children aged 12-23 months in Ethiopia.

Adequate ANC visits were the top-ranked attribute to predict childhood among children aged 12-23 months in Ethiopia, with a 0.087 information gain value. This finding was agreed upon with the previous similar studies done in Ethiopia [14, 68], and Zimbabwe [69]. This might be due to women who attend ANC follow-up might get counseling about child immunization [70], and mothers might receive adequate education about the importance of postnatal visits and activities [71]. Institutional delivery was the second-most important attribute to predict childhood vaccination. This finding is supported by similar studies done in Ethiopia [8, 14], and Nigeria [72]. This might be because some vaccines such as, BCG and OPV 0 are often given immediately after birth at health facilities [70].

Visiting HF was the third most important attribute to predict childhood vaccination in Ethiopia. This finding was in line with studies done in Ethiopia [14], and similar resource-limited settings [73, 74]. This might be due to the fact that mothers who visit a health facility might receive

adequate education and counseling about child immunization, and mothers after birth are recommended to visit a health facility for postnatal check-ups and services.

Higher educational status of mothers was the fourth important attributes to predict childhood vaccination among children aged 12-23 months. This study also similar with study done in Bangladesh that maternal education are important features to predict anemia among under five years of children [75]. Another study done in India also support this finding of the study [76]. This might be due to educated mothers might know the importance of vaccine for child care, and educated mothers might empower them and feel free to make decisions to visit the health facility for child health services [77]. Being rich and urban resident were the fifth and sixth important attribute to predict childhood vaccination among children aged 12-23 months in Ethiopia. This finding was similar with study done in Bangladesh [75], and Ethiopia [8]. This might be because mothers from Urban areas might have more access to media, which play a very vital role in disseminating educational information and creating awareness [78, 79]. Therefore, children in urban areas are more likely to uptake vaccines.

Generating rules for childhood vaccination was the third objective of the study. Consequently, seven association rules were generated to determine vaccination status among children aged 12-23 months in Ethiopia. According to association rule 1, the probability of a childhood vaccination would be 86.73%, if and only if mothers' wealth status was rich, mothers had adequate ANC visits, and children were urban residents. This might be because women with rich wealth status might be able to afford to pay any costs needed for vaccination, mothers who had adequate ANC visits might had adequate awareness and knowledge about child vaccination during their health facility visits during their pregnancy period, and health facilities in urban areas might be easily accessible for mothers to vaccinate their children. The effects of these three attributes are critical for childhood vaccination, and the combination of these factors might make it particularly important for children to be vaccinated when they were under 12 to 23 months. Based on Rule 2, childhood vaccination would be 82.14%, if mothers gave birth at a health institution, children were from higher-educated mothers, and if the household head was female. The if/ then rules are critical to discovering hidden relationships between attributes, extracting knowledge from a set of data, and accurately representing knowledge and information about vaccination of children. The findings presented in this study are critically important for policymakers and stakeholders to support public health action, decision-making purposes, and the storage of knowledge regarding child vaccination status.

Strengths and limitation of the study

In this study, machine learning algorithms are used to classify, and predict childhood vaccination. This study used nationally representative data, and the findings might be representative for the study populations. However, machine learning algorithms do not have coefficients like odds and incident rate ratios. Therefore, the strength and direction of associations are unknown.

Conclusions

This study was aimed at evaluating and identifying the best machine learning algorithms to classify and select important attributes to predict childhood vaccination among children aged 12-23 months in Ethiopia. Based on the objectives, eight machine learning algorithms were considered to accurately predict childhood vaccination. Different confusion matrix elements were used to compare the candidate machine learning algorithms. Based on the results, PART, J48, multilayer perceptron, and random forest algorithms were the first, second, third, and fourth best performing algorithms to predict childhood vaccination among children aged 12-23 months in Ethiopia. Attributes such as an adequate ANC visit, institutional delivery, visits to health facilities, the higher educational status of mothers, and the rich wealth status of mothers were the top five important attributes to predict childhood vaccination among children aged 12-23 months in Ethiopia.

Generating association rules for childhood vaccination was another objective of the study. Accordingly, seven rules were generated that are associated with the vaccination of children aged 12-23 months. The findings of this study would support policymakers and stakeholders in developing child care intervention mechanisms and early preparedness for caring for children through child immunization. This study serves as input for immunization coverage and reduction of vaccine dropouts. The generated rule is also important for knowledge extraction and knowledge representation.

Abbreviations

ANC: Antenatal care, **BCG:** Bacillus Calmette Guerin, **EDHS:** Ethiopian Demographic and Health survey, **AUC:** Area under recursive curve, **WHO:** World health organization, **DTP:** Diphtheria Pertussis Tetanus.

Declarations

Consent for publication

Not applicable.

Availability of data and materials

The dataset used for analysis is available on the DHS program website. All the data generated and analyzed are included in the study.

Patient and public participation

Not applicable.

Competing interests

The author declared that there are no competing interests in this work.

Funding

No funding was received.

Author contributions

AWD had substantial contributions to the study design, conception, data analysis, interpretation and discussion of the findings. The author had substantial contributions in drafting and revising the manuscript, and the author had read and approved the final manuscript for submission.

Acknowledgment

The author would like to express deepest appreciation to the DHS program for giving permission to access and use the data for this study.

Reference

1. Unicef, *statistical snapshot. Child mortality: Accessed from* https://data.unicef.org/resources/2013-statistical-snapshot-child-mortality/. New York, 2013.
2. Organization, W.H., *Meeting report: WHO technical consultation: nutrition-related health products and the World Health Organization model list of essential medicines–practical considerations and feasibility: Geneva, Switzerland, 20–21 September 2018*. 2019, World Health Organization.
3. UNICEF and W.H. Organization, *Levels & trends in child mortality estimates developed by the UN Inter-Agency Group for Child Mortality Estimation.* 2015.

4. Sakelo, A.N., et al., *Newborn care practice and associated factors among mothers of one-month-old infants in Southwest Ethiopia.* International journal of pediatrics, 2020. **2020**: p. 1-7.
5. Organization, W.H., *World health statistics 2016: monitoring health for the SDGs sustainable development goals*. 2016: World Health Organization.
6. Meleko, A., M. Geremew, and F. Birhanu, *Assessment of child immunization coverage and associated factors with full vaccination among children aged 12–23 months at Mizan Aman town, bench Maji zone, Southwest Ethiopia.* International journal of pediatrics, 2017. **2017**.
7. WHO, U., *World Bank. State of the World's Vaccines and Immunization. Geneva, Switzerland: World Health Organization; 2009: Accessed from* https://www.tandfonline.com/doi/abs/10.4161/hv.6.2.11326. 2010.
8. Tesfaye, T.D., W.A. Temesgen, and A.S. Kasa, *Vaccination coverage and associated factors among children aged 12–23 months in Northwest Ethiopia.* Human vaccines & immunotherapeutics, 2018. **14**(10): p. 2348-2354.
9. Touray, E., et al., *Childhood vaccination uptake and associated factors among children 12–23 months in rural settings of the Gambia: a community-based cross-sectional study.* BMC Public Health, 2021. **21**(1): p. 1-10.
10. Taiwo, L., et al., *Factors affecting access to information on routine immunization among mothers of under 5 children in Kaduna State Nigeria, 2015.* Pan African Medical Journal, 2017. **27**(1).
11. *WHO, Vaccines and immunization*. 2023. https://www.who.int/health-topics/vaccines-and-immunization#tab=tab_1.
12. Payne, S., et al., *Achieving comprehensive childhood immunization: an analysis of obstacles and opportunities in The Gambia.* Health policy and planning, 2014. **29**(2): p. 193-203.
13. Ndiritu, M., et al., *Immunization coverage and risk factors for failure to immunize within the Expanded Programme on Immunization in Kenya after introduction of new Haemophilus influenzae type b and hepatitis b virus antigens.* BMC public health, 2006. **6**(1): p. 1-8.
14. Dirirsa, K., et al., *Assessment of vaccination timeliness and associated factors among children in Toke Kutaye district, central Ethiopia: A Mixed study.* Plos one, 2022. **17**(1): p. e0262320.
15. Chandir, S., et al., *Using predictive analytics to identify children at high risk of defaulting from a routine immunization program: feasibility study.* JMIR public health and surveillance, 2018. **4**(3): p. e9681.
16. Animaw, W., et al., *Expanded program of immunization coverage and associated factors among children age 12–23 months in Arba Minch town and Zuria District, Southern Ethiopia, 2013.* BMC public health, 2014. **14**(1): p. 1-10.
17. Collishaw, N.E., *The millennium development goals and tobacco control.* Global Health Promotion, 2010. **17**(1_suppl): p. 51-59.
18. Debie, A. and B. Taye, *Assessment of fully vaccination coverage and associated factors among children aged 12–23 months in Mecha District, north West Ethiopia: a cross-sectional study.* Sci J Public Health, 2014. **2**(4): p. 342-8.
19. Mohammed, H. and A. Atomsa, *Assessment of child immunization coverage and associated factors in Oromia regional state, eastern Ethiopia.* Science, Technology and Arts Research Journal, 2013. **2**(1): p. 36-41.
20. Negussie, A., et al., *Factors associated with incomplete childhood immunization in Arbegona district, southern Ethiopia: a case–control study.* BMC public health, 2015. **16**(1): p. 1-9.
21. Ekouevi, D.K., et al., *Incomplete immunization among children aged 12–23 months in Togo: a multilevel analysis of individual and contextual factors.* BMC public health, 2018. **18**: p. 1-10.
22. Budu, E., et al., *Trend and determinants of complete vaccination coverage among children aged 12-23 months in Ghana: analysis of data from the 1998 to 2014 Ghana demographic and health surveys.* Plos one, 2020. **15**(10): p. e0239754.

23. Tegene, T., et al., *Newborn care practice and associated factors among mothers who gave birth within one year in Mandura District, Northwest Ethiopia.* Clinics in Mother and Child Health, 2015. **12**(1).
24. Pepe, M.S., et al., *Limitations of the odds ratio in gauging the performance of a diagnostic, prognostic, or screening marker.* American journal of epidemiology, 2004. **159**(9): p. 882-890.
25. Saroj, R.K., et al., *Machine Learning Algorithms for understanding the determinants of under-five Mortality.* BioData mining, 2022. **15**(1): p. 1-22.
26. Cody, S. and A. Asher, *Smarter, better, faster: The potential for predictive analytics and rapid-cycle evaluation to improve program development and outcomes*. 2014, Mathematica Policy Research.
27. Cheong, Q., et al., *Predictive modeling of vaccination uptake in US counties: A machine learning–based approach.* Journal of medical Internet research, 2021. **23**(11): p. e33231.
28. Mannion, N., *Predictions of changes in child immunization rates using an automated approach: USA*. 2020, Dublin, National College of Ireland.
29. Tesfaye, B., et al., *Determinants and development of a web-based child mortality prediction model in resource-limited settings: a data mining approach.* Computer methods and programs in biomedicine, 2017. **140**: p. 45-51.
30. Osisanwo, F., et al., *Supervised machine learning algorithms: classification and comparison.* International Journal of Computer Trends and Technology (IJCTT), 2017. **48**(3): p. 128-138.
31. Jaskari, J., et al., *Machine learning methods for neonatal mortality and morbidity classification.* Ieee Access, 2020. **8**: p. 123347-123358.
32. Fenta, H.M., T. Zewotir, and E.K. Muluneh, *A machine learning classifier approach for identifying the determinants of under-five child undernutrition in Ethiopian administrative zones.* BMC Medical Informatics and Decision Making, 2021. **21**(1): p. 1-12.
33. Thangamani, D. and P. Sudha, *Identification of malnutrition with use of supervised datamining techniques–decision trees and artificial neural networks.* Int J Eng Comput Sci, 2014. **3**(09).
34. Kuttiyapillai, D. and R. Ramachandran, *Improved text analysis approach for predicting effects of nutrient on human health using machine learning techniques.* IOSR J Comput Eng, 2014. **16**(3): p. 86-91.
35. Dhar, A., N.S. Dash, and K. Roy. *An innovative method of feature extraction for text classification using part classifier*. in *Information, Communication and Computing Technology: Third International Conference, ICICCT 2018, New Delhi, India, May 12, 2018, Revised Selected Papers 3*. 2019. Springer.
36. *The 2016 Ethiopian Demography and Health Survey*. https://dhsprogram.com/methodology/survey/survey-display-478.cfm.
37. Wakeyo, M.M., et al., *Short birth interval and its associated factors among multiparous women in Mieso agro-pastoralist district, Eastern Ethiopia: A community-based cross-sectional study.* Front Glob Womens Health, 2022. **3**: p. 801394.
38. Demsash, A.W., et al., *Spatial distribution of vitamin A rich foods intake and associated factors among children aged 6–23 months in Ethiopia: spatial and multilevel analysis of 2019 Ethiopian mini demographic and health survey.* BMC nutrition, 2022. **8**(1): p. 1-14.
39. Muhwava, L.S., N. Morojele, and L. London, *Psychosocial factors associated with early initiation and frequency of antenatal care (ANC) visits in a rural and urban setting in South Africa: a cross-sectional survey.* BMC pregnancy and childbirth, 2016. **16**(1): p. 1-9.
40. Demsash, A.W., et al., *Spatial distribution of vitamin A rich foods intake and associated factors among children aged 6–23 months in Ethiopia: spatial and multilevel analysis of 2019 Ethiopian mini demographic and health survey.* BMC Nutrition, 2022. **8**(1): p. 77.
41. Levy, P.S. and S. Lemeshow, *Sampling of populations: methods and applications*. 2013: John Wiley & Sons.
42. Webb, G.I., E. Keogh, and R. Miikkulainen, *Naïve Bayes.* Encyclopedia of machine learning, 2010. **15**: p. 713-714.

43. Hall, M., et al., *The WEKA data mining software: an update.* ACM SIGKDD explorations newsletter, 2009. **11**(1): p. 10-18.
44. Hosmer Jr, D.W., S. Lemeshow, and R.X. Sturdivant, *Applied logistic regression*. Vol. 398. 2013: John Wiley & Sons.
45. Uddin, S., et al., *Comparing different supervised machine learning algorithms for disease prediction.* BMC medical informatics and decision making, 2019. **19**(1): p. 1-16.
46. Kaur, G. and A. Chhabra, *Improved J48 classification algorithm for the prediction of diabetes.* International journal of computer applications, 2014. **98**(22).
47. Sharma, A.K. and S. Sahni, *A comparative study of classification algorithms for spam email data analysis.* International Journal on Computer Science and Engineering, 2011. **3**(5): p. 1890-1895.
48. Saroj, R.K., et al., *Machine Learning Algorithms for understanding the determinants of under-five Mortality.* BioData Min, 2022. **15**(1): p. 20.
49. Fu, G., X. Dai, and Y. Liang, *Functional random forests for curve response.* Sci Rep, 2021. **11**(1): p. 24159.
50. Tkachev, V., et al., *Flexible Data Trimming Improves Performance of Global Machine Learning Methods in Omics-Based Personalized Oncology.* Int J Mol Sci, 2020. **21**(3).
51. Yu, Y., et al., *Machine Learning Methods for Predicting Long-Term Mortality in Patients After Cardiac Surgery.* Front Cardiovasc Med, 2022. **9**: p. 831390.
52. *What is AdaBoost Algorithm Model?: Accessed from* https://data-flair.training/blogs/adaboost-algorithm/.
53. Kamarudin, M.H., et al., *A logitboost-based algorithm for detecting known and unknown web attacks.* IEEE Access, 2017. **5**: p. 26190-26200.
54. Narkhede, S., *Understanding auc-roc curve.* Towards Data Science, 2018. **26**(1): p. 220-227.
55. El Khouli, R.H., et al., *Relationship of temporal resolution to diagnostic performance for dynamic contrast enhanced MRI of the breast.* Journal of Magnetic Resonance Imaging: An Official Journal of the International Society for Magnetic Resonance in Medicine, 2009. **30**(5): p. 999-1004.
56. McHugh, M.L., *Interrater reliability: the kappa statistic.* Biochem Med (Zagreb), 2012. **22**(3): p. 276-82.
57. Molnar, C., *Interpretable machine learning*. 2020: Lulu. com.
58. Shi, R., et al., *Obesity is negatively associated with dental caries among children and adolescents in Huizhou: a cross-sectional study.* BMC Oral Health, 2022. **22**(1): p. 76.
59. Ivančević, V., et al., *Using association rule mining to identify risk factors for early childhood caries.* Computer methods and programs in biomedicine, 2015. **122**(2): p. 175-181.
60. Zafar, A., et al., *Machine learning-based risk factor analysis and prevalence prediction of intestinal parasitic infections using epidemiological survey data.* PLOS Neglected Tropical Diseases, 2022. **16**(6): p. e0010517.
61. Tandan, M., et al., *Discovering symptom patterns of COVID-19 patients using association rule mining.* Computers in biology and medicine, 2021. **131**: p. 104249.
62. Li, Q., et al., *Mining association rules between stroke risk factors based on the Apriori algorithm.* Technology and Health Care, 2017. **25**(S1): p. 197-205.
63. Kebede, S.D., et al., *Prediction of contraceptive discontinuation among reproductive-age women in Ethiopia using Ethiopian Demographic and Health Survey 2016 Dataset: A Machine Learning Approach.* BMC Medical Informatics and Decision Making, 2023. **23**(1): p. 1-17.
64. Bitew, F.H., et al., *Machine learning approach for predicting under-five mortality determinants in Ethiopia: evidence from the 2016 Ethiopian Demographic and Health Survey.* Genus, 2020. **76**: p. 1-16.
65. Fenta, H.M., et al., *Determinants of stunting among under-five years children in Ethiopia from the 2016 Ethiopia demographic and Health Survey: Application of ordinal logistic regression*

model using complex sampling designs. Clinical Epidemiology and Global Health, 2020. **8**(2): p. 404-413.

66. Talukder, A. and B. Ahammed, *Machine learning algorithms for predicting malnutrition among under-five children in Bangladesh.* Nutrition, 2020. **78**: p. 110861.
67. Kassie, G.W. and D.L. Workie, *Determinants of under-nutrition among children under five years of age in Ethiopia.* BMC Public Health, 2020. **20**(1): p. 1-11.
68. Tesfa, G.A., et al., *Spatial distribution and associated factors of measles vaccination among children aged 12–23 months in Ethiopia. A spatial and multilevel analysis.* Human Vaccines & Immunotherapeutics, 2022. **18**(1): p. 2035558.
69. Mukungwa, T., *Factors associated with full immunization coverage amongst children aged 12–23 months in Zimbabwe.* African Population Studies, 2015. **29**(2).
70. Tamirat, K.S. and M.M. Sisay, *Full immunization coverage and its associated factors among children aged 12–23 months in Ethiopia: further analysis from the 2016 Ethiopia demographic and health survey.* BMC public health, 2019. **19**: p. 1-7.
71. Gualu, T. and A. Dilie, *Vaccination coverage and associated factors among children aged 12–23 months in debre markos town, Amhara regional state, Ethiopia.* Advances in Public Health, 2017. **2017**.
72. Antai, D., *Regional inequalities in under-5 mortality in Nigeria: a population-based analysis of individual-and community-level determinants.* Population health metrics, 2011. **9**: p. 1-10.
73. Darroch, J.E., G. Sedgh, and H. Ball, *Contraceptive technologies: responding to women's needs.* New York: Guttmacher Institute, 2011. **201**(1): p. 1-51.
74. Kozuki, N. and N. Walker, *Exploring the association between short/long preceding birth intervals and child mortality: using reference birth interval children of the same mother as comparison.* BMC public health, 2013. **13**(3): p. 1-10.
75. Khan, J.R., et al., *Machine learning algorithms to predict the childhood anemia in Bangladesh.* Journal of Data Science, 2019. **17**(1): p. 195-218.
76. Khare, S., et al., *Investigation of nutritional status of children based on machine learning techniques using Indian demographic and health survey data.* Procedia computer science, 2017. **115**: p. 338-349.
77. Bekele, Y.A. and G.A. Fekadu, *Factors associated with HIV testing among young females; further analysis of the 2016 Ethiopian demographic and health survey data.* PLoS One, 2020. **15**(2): p. e0228783.
78. Oginni, A.B., S.B. Adebajo, and B.A. Ahonsi, *Trends and determinants of comprehensive knowledge of HIV among adolescents and young adults in Nigeria: 2003-2013.* African journal of reproductive health, 2017. **21**(1): p. 26-34.
79. Haque, M.A., et al., *Factors associated with knowledge and awareness of HIV/AIDS among married women in Bangladesh: evidence from a nationally representative survey.* SAHARA-J: Journal of Social Aspects of HIV/AIDS, 2018. **15**(1): p. 121-127.
