# Supplementary material for: Machine learning algorithms’ application to predict childhood vaccination among children aged 12–23 months in Ethiopia: Evidence 2016 Ethiopian Demographic and Health Survey dataset
Source: PLoS One. 2023 Oct 18;18(10):e0288867. doi: 10.1371/journal.pone.0288867 (PMC10584162; doi:10.1371/journal.pone.0288867)
Supplement: Attachment — Submitted filename: Point-by-poinr-response.docx [file pone.0288867.s003.docx]

**Dear Editors of Plos one journal**

The author has been recalled to revised the manuscript, originally entitled as ***Machine learning algorithms’ application to predict childhood vaccination among children aged 12-23 months in Ethiopia: Evidence 2016 Ethiopian demographic and health survey dataset,*** and identified by a manuscript number PONE-D-23-09622 under Plos one Journal, which was submitted for publication. I have received the editor(s)’ and reviewers’ comment for the advancement of the manuscript.

Thank you for giving a chance to revise the manuscript. I really appreciate the editors, reviewers for taking time to review the manuscript and give detailed and constructive comments, which has great importance to improve/ strengthen the manuscript.

Finally, all the required documents in their revised form have been summited. I hope that I have addressed all the questions and if you have any points for further clarity, please let me know.

**Thank so much editor (s) and reviewers**

**Correspondence author**

**On behalf of all aythors!**

**Point by point response to editor (s) and reviewers’ comments**

**Comments to the Author**

1. Is the manuscript technically sound, and do the data support the conclusions?

The manuscript must describe a technically sound piece of scientific research with data that supports the conclusions. Experiments must have been conducted rigorously, with appropriate controls, replication, and sample sizes. The conclusions must be drawn appropriately based on the data presented.

Reviewer #1: Yes

Reviewer #2: Yes

**Author:** Thank you! Too much respect.

2. Has the statistical analysis been performed appropriately and rigorously?

Reviewer #1: Yes

Reviewer #2: Yes

**Author:** Thank you! Too much respect.

3. Have the authors made all data underlying the findings in their manuscript fully available?
Reviewer #1: Yes

Reviewer #2: Yes

**Author:** Thank you! Too much respect.

4. Is the manuscript presented in an intelligible fashion and written in standard English?
Reviewer #1: Yes

Reviewer #2: Yes

**Author:** Thank you! Too much respect.

5. Review Comments to the Author
Please use the space provided to explain your answers to the questions above. You may also include additional comments for the author, including concerns about dual publication, research ethics, or publication ethics. (Please upload your review as an attachment if it exceeds 20,000 characters)

**Reviewer #1:** This type of methodological modeling research in operational research is not as such common and I appreciate the authors to come up with such insightful new approach.
Few concerns and suggestion for the authors: Q1? It was better if you make the presentation those statistical modelling easier for all readers to read.

**Author:** Thank you so much for your motivational message! Revisions has made on the statistical model for easy to read**.
Reviewer #1:** Q2? Through this machine learning algorithms approach of identifying predictors of child vaccination, what is new from the usual operational research approach.

**Author:** Thank you so much again! As you know, in the usual operational research adjusted odds ratio and relative risk with some level of confidence interval used to determine statistical association of variables. For instance, variable X may be statistically significant at 95% CI for the target variable W, but Y may not be significant at 95% CI for target variable W. If we set our confidence interval to 90% and 99%, then two things may happen. At 90% CI X may not be significant and at 99% CI Y may be found to be significant. Plus, researchers usually select the variable with p-value <=0.2 and <=0.05 for bivariate and multivariate analysis to know their strength of association, respectively. This may loss variable with a p-value of 0.21, 0.22, 0.23, 0.24, and 0.051,0.052…0.054, which are nearly the same with p-value of 0.2 and 0.05, at bivariate and multivariate analysis, respectively. Here, we can understand two things, the significance of the variable might be caused by **(1)** confidence interval, and **(2)** cut point of the p-value. In machine learning algorithms, these problems are not found. Simply, each independent variable might have their own contribution for the target variable to happen/ not happen, and their contribution is ordered by the information gain value. In general machine learning algorithms are very important to handle huge, and various attribute and records. The algorithms are critical to discover new insight and important to know how much the independent variables jointly determine the target variable (association rules).
**Reviewer #2:** Are you predicting vaccination uptake or whether the children have taken vaccinations? Or the outcome of the vaccinations? Why do we predict? Is there no other means to gain this data?

**Author:** Thank you for your interesting questions! As you know, the outcome of interest is childhood vaccination. For us, vaccination uptake is similar with childhood vaccination or it might be different due to researchers’ operationalization. Though the outcome is clearly presented in the manuscript, for the purpose of clarification, It is about predicting childhood vaccination among children aged 12-23 months. Childhood vaccination is when children aged 12-23 months take at least one dose of BCG, three doses of DPT, three dose of polio, and one dose of measles vaccine. Prediction is one way of research analysis method, so it is very important for identifying predicators by their order of information gain value through using best performance algorithm. It is also critical to generate association rules that variable jointly determine the outcome of interest. Therefore, the finding is also serving as an input for stakeholder preparedness, setting intervention, and critical for theoretical learning and practical practice. According to our evidence, and Ethiopian demographic and health survey report, there are three ways that used to gain the data such as (1) written vaccination record that includes infant immunisation card and other health cards, (2) the mothers’ verbal reports, and (3) health facility records. **Reviewer #2:** The abstract section needs major revision E.g., Mixed reporting in the methods and result section and the key terms are incomplete

**Author:** Thank you so much! I have made revisions in the abstract and made distinct it with the result. Key terms were included and modified.

**Reviewer #2:** Which model was used? It is inconsistent in the methods and results section.

**Author:** Thank you so much! As you know, I have considered eight machine-learning algorithms for model-building and comparison purposes. After comparison, the best machine learning algorithm (PART) was selected, then important attributes for childhood vaccination were selected based on the best algorithm (PART) according to this study. I have used revised the abstract and made a distinction with the result. Thank you again for your constructive comments.

**Reviewer #2:** Shorten and add efforts made for improving vaccination in Ethiopia.

**Author:** Thank you so much! Revisions have been made on the outcome variable and I focused on Ethiopia for discussing and justifying the finding.
**Reviewer #2:** The study area is NOT referenced.

**Author:** Thank you so much! I have cited the study area, now.
**Reviewer #2:** In the operational definitions try to be specific E.g., is that access to media or use of the media?

**Author:** Thank you so much again! It is about media access. As you know, if the mothers had media access, the mothers are more likely to be exposed for varieties of information.

**Reviewer #2:** In the model building section appropriate references are lacking

**Author:** You are right that appropriate references are limited! This is because there are no sufficient similar studies regarding the current study. So, I have written it as a limitation.
**Reviewer #2:** You can rewrite “Children’s and mothers’ characteristics” for example as “Socio-demographic Characteristics of Children’s and mothers” and avoid inconsistency e.g., basic characteristics….

**Author:** Thank you! I have written it shortly and consistently, based on your feedback.
**Reviewer #2:** The tables and figures are not self-explanatory. In addition, the figure is not referenced.

**Author:** Thank you very much for your critical review! I have made the table and figure as possible to be self-explanatory, and the figures have been referenced in their correct order.

**Reviewer #2:** Try to see this sentence again “This might be because some vaccines such as BCG and OPV 0 are often 460 given immediately after birth at health facilities [70].” With the percentage of institutional delivery. Similarly, try to revisit “household head 491 was female” because it is very low and is associated with your statistical efficiency.

**Author:** Thank you so much for your critical feedback! I have made improvement on it.
**Reviewer #2:** The result section needs brief revision.

**Author:** Thank you so much! Revision has made in the result sections.

**Reviewer #2:** The discussion section is inadequately discussed and reasoned out.

**Author:** Thank you so much! Now, discussions have been revised and justified.
**Reviewer #2:** The recommendations and the conclusions should be context based and practical.

**Author:** Thank you so much! I have written the conclusion shortly based on the findings, and recommendation that will support for practical, theoretical, and future research direction were forwarded clearly.
**Reviewer #2:** For your research carrier try to do with others/team which is one component of professionalism

**Author:** Thank you so much! I will do with others, especially with my colleagues.
